# TFAM Expression, rs1937 Variant, and Mitochondrial DNA Copy Number in Egyptian Myocardial Infarction Patients

**DOI:** 10.3390/ijms27219617

**Published:** 2026-10-27

**Authors:** Sherin Sobhy El naidany, Heba F. Khader, Asmaa Abdelkarim Kenawy, Mohammed G. Elhelbawy, Zahraa Azab Mohamed Abouelatta, Hoda Ahmed Abdelmoneim, Reham Abdulla Aboukhalil, Wael Tawfik, Mai A. H. Abouelenin

**Affiliations:** 1 Medical Biochemistry and Molecular Biology Department, Faculty of Medicine, Menoufia University, Shebin El Kom 32511, Egypt; sherinsobhy@ymail.com (S.S.E.n.); hf.khader79@gmail.com (H.F.K.); 2 Department of Basic Medical Sciences, Unaizah College of Medicine and Medical Sciences, Qassim University, Unaizah 51452, Saudi Arabia; 3 Cardiology Department, Faculty of Medicine, Menoufia University, Shebin El Kom 32511, Egypt; asmaa.kenawy@med.menofia.edu.eg; 4 Clinical Pathology Department, Faculty of Medicine, Menoufia University, Shebin El Kom 32511, Egypt; mohammadhelbawy@yahoo.com (M.G.E.); zahraa.mohamed@med.menofia.edu.eg (Z.A.M.A.); 5 Medical Biochemistry and Molecular Biology Department, Faculty of Medicine, Cairo University, Cairo 11956, Egypt; hodaghonem888@hotmail.com; 6 Department of Pathology, College of Medicine, Qassim University, Unaizah 51452, Saudi Arabia; re.aboukhalil@qu.edu.sa; 7 Cardiology Department, Faculty of Medicine, Benha University, Benha 13518, Egypt; wtwfik@hotmail.com

**Keywords:** MI, *TFAM*, polymorphism, mitochondrial DNA CN

## Abstract

Myocardial infarction (MI) is a fatal condition that causes damage to the heart muscle by abruptly stopping blood flow and oxygenation. Mitochondrial transcription factor A (*TFAM*) and mitochondrial DNA copy number (mtDNA CN) show an increased rate of association with development of several human diseases and malignancies. This paper, therefore, aims to identify the possible relation among mtDNA CN and its regulating gene *TFAM* (expression of the gene and rs1937 polymorphism) as markers in myocardial infarction among Egyptians. A total of 200 participants were enrolled and divided into two groups. Group 1 included 100 myocardial infarction patients with ST-segment elevation (STEMI) recruited from from the Coronary Care Unit (CCU) of Menoufia University Hospitals, while group 2 included 100 Controls. Blood samples were collected from all participants immediately after diagnosis of MI and before beginning therapy. *TFAM* SNP (rs1937) was determined by the allelic discrimination TaqMan technique, and mtDNA CN and *TFAM* gene expression were detected using real-time quantitative PCR in 100 MI patients and 100 controls. *TFAM* mRNA expression and mtDNA copy number were profoundly reduced in MI patients than controls. Analysis of genotype showed that the *TFAM* rs1937 variant distribution was in Hardy-Weinberg equilibrium. The C allele and the GC genotype were substantially more frequent in the patients’ cohort (*p* = 0.006 and 0.005, respectively). The GC genotype of the *TFAM* polymorphism (rs1937) showed a preliminary association with MI in STEMI patients. Limitations of this study are the absence of longitudinal pre-MI or post-recovery data. *TFAM* mRNA expression and mtDNA copy number were measured in whole blood without adjustment for leukocyte subpopulations or platelet counts. Small number of C carriers of TFAM rs1937 variant and absence of CC genotype limited statistical power and requires replication in larger Egyptian cohort. These findings suggest that decreased *TFAM* gene expression and mtDNA CN were significant biomarkers associated with acute myocardial infarction in STEMI patients.

## 1. Introduction

Coronary artery disease (CAD) is one of the main reasons for death throughout the world [1]. Myocardial infarction (MI) is a severe disorder that can be fatal and is usually heart-related [2]. One effective way of decreasing complicated cases, as well as fatal outcomes, is the prevention and control of the condition via the dissemination of information about the associated risk factors [1]. Hyperlipidemia, hypertension, smoking, obesity, diabetes mellitus (DM), and atherosclerosis family history are considered risk factors for MI [3]. Genetic variables are important contributors to the onset and course of MI [4].

Most of these risk factors would cause an abundance of reactive oxygen species (ROS), which would aid in the atherosclerotic process. Several studies have recently revealed how mitochondrial dysfunction contributes to the pro-atherogenic impact of the primary cardiovascular risk factors [5]. Mitochondrial DNA copy number (mtDNA CN), which is the ratio of mitochondrial DNA to nuclear DNA, is a principal indicator of how many mitochondria are present and how well they are functioning. It can also give us an idea of how much damage the mtDNA has sustained [6].

Different factors, including mtDNA mutations and genetic variants of genes involved in mitochondrial DNA (mtDNA) synthesis and transcription, can affect the control of the number of mtDNA copies [7]. mtDNA CN in blood or tissue has been consistently linked to all-cause mortality and a spectrum of age-related pathologies, like cancer, chronic kidney disease, and cardiovascular disorders [8,9].

Mitochondria have another important function in that they are a source of reactive oxidative species (ROS), as well as providing most of the energy in the cell. The mitochondrial integrity, however, is often lost in situations where there is an ischemia-reperfusion (IR) injury. This mitochondrial injury is a principal mechanism underlying cardiac and other tissue injuries, initiated by energy stress and an abundance of ROS. This cascade leads to oxidative stress, increased intracellular calcium, and both necrotic and apoptotic cell-death pathways [10].

Damaged mitochondria actively contribute to the development of an inflammatory milieu by initiating important signaling pathways. In this way, mitochondria have an ambidextrous role where they can either contribute to or regulate inflammation [11]. Cardioprotective strategies, however, have constantly shown that mitochondria are among the main targets for decreasing cell death, which results in left ventricular dysfunction and eventually leads to heart failure [12]. Mitochondrial transcription factor A (*TFAM*) is essential for mitochondrial DNA replication, transcription, and nucleoid compaction, all of which preserve the integrity and functionality of mitochondria. TFAM is a nuclear-encoded protein that is highly conserved. It affects mitochondrial homeostasis and cellular energy consumption and is essential for mitochondrial biogenesis. Because TFAM is essential for mitochondrial function, its malfunction leads to the development of several diseases [13]. TFAM has a major role in keeping mtDNA CN steady and is also involved in the regulation of the inflammatory process and control of the mitochondrial genome’s activity [14]. Previous studies have revealed a link between polymorphisms of the TFAM gene and inflammatory diseases, like Alzheimer’s disease, Huntington’s disease-associated neuropathy [15], and diabetic neuropathy [16]. The single nucleotide polymorphism rs1937 (G > C) is located in exon 1 of TFAM gene and encodes a non-synonymous Ser12Thr (S12T) substitution in the mitochondrial targeting sequence of the protein. This variant has been previously associated with mitochondrial dysfunction-related disorders [17]. Our study aims to assess the role of mitochondrial DNA CN, *TFAM* gene expression, and *TFAM* rs1937 variant in myocardial infarction in the Egyptian population.

## 2. Results

The demographic and clinical characteristics of the 200 participants, 100 MI patients, and 100 controls are outlined in Table 1. Controls were recruited with a similar age distribution. There was no statistically significant difference in age and sex between groups (*p* > 0.05, small effect size). BMI, however, was substantially higher in the group of MI patients compared to the controls representing large effect size (*p* < 0.001, Cohen’s d = 0.676). Smoking status was not substantially different between the two groups (*p* = 0.067). Among the MI patients, 50% (*n* = 50) had hypertension and 37% (*n* = 37) had diabetes mellitus.

Substantial differences were revealed between patients and controls in terms of laboratory markers and genetic features. Cardiac biochemical indicators (Troponin I, CK-MB), as well as blood cholesterol profiles (TC, LDL-c), were profoundly higher in the patient group and showed large effect sizes (r = 0.864, r = 0.811, Cohen’s d = 1.592, and Cohen’s d = 1.377, respectively, whereas the HDL-c level was markedly lower (*p* < 0.001 for all and 0.002 for HDL.c). The TG levels, however, did not greatly differ between the two groups (*p* = 0.854). At the molecular level, *TFAM* mRNA expression and mtDNA CN were substantially lower in patients (0.58 ± 0.15 and 0.50 ± 0.18, respectively) than in controls (1.04 ± 0.12 and 1.10 ± 0.17, respectively; *p* < 0.001) with a very large effect size (Cohen’s d = −3.432, 95% CI: −3.867–−2.993 for *TFAM* expression indicating almost non-overlapping distributions) and r = 0.857, 95% CI: 0.815–0.890 for mtDNA CN). After applying the Benjamini–Hochberg false discovery rate (FDR) correction, all statistically significant associations remained significant. (Table 1).

Table 2 shows the distribution of *TFAM* rs1937 genotypes. The observed genotype frequencies were in the Hardy-Weinberg equilibrium in each of the studied groups (*p* > 0.05). The GC genotype and the C allele had a noticeably greater frequency in MI patients compared to controls (16% vs. 4%; *p* = 0.005 and 0.006, respectively). This association corresponded with a 95% CI of 1.471–14.210 for the GC genotype and 95% CI: 1.399–12.981 for the C allele, with odds ratios of 4.571 and 4.261, respectively.

To address the potential influence of the marked differences in hypertension and diabetes mellitus between cases and controls, we performed an additional sensitivity analysis restricted to participants without either hypertension or diabetes mellitus. This analysis included 36 MI patients and 100 controls. *TFAM* mRNA expression and mtDNA copy number remained markedly different between MI patients and controls (both *p* < 0.001), whereas the distribution of the rs1937 genotype was no longer significantly different (Fisher’s exact *p* = 0.381). These findings support the robustness of the associations of *TFAM* expression and mtDNA copy number with MI after eliminating hypertension and diabetes mellitus as potential confounding factors. However, the rs1937 finding should be interpreted cautiously as a preliminary association because only three GC carriers remained among the restricted MI cases. We acknowledge that this sensitivity analysis reduces, but does not completely eliminate, the possibility of residual confounding arising from the selection of comparatively healthy controls (Table 3).

**Figure 1:**
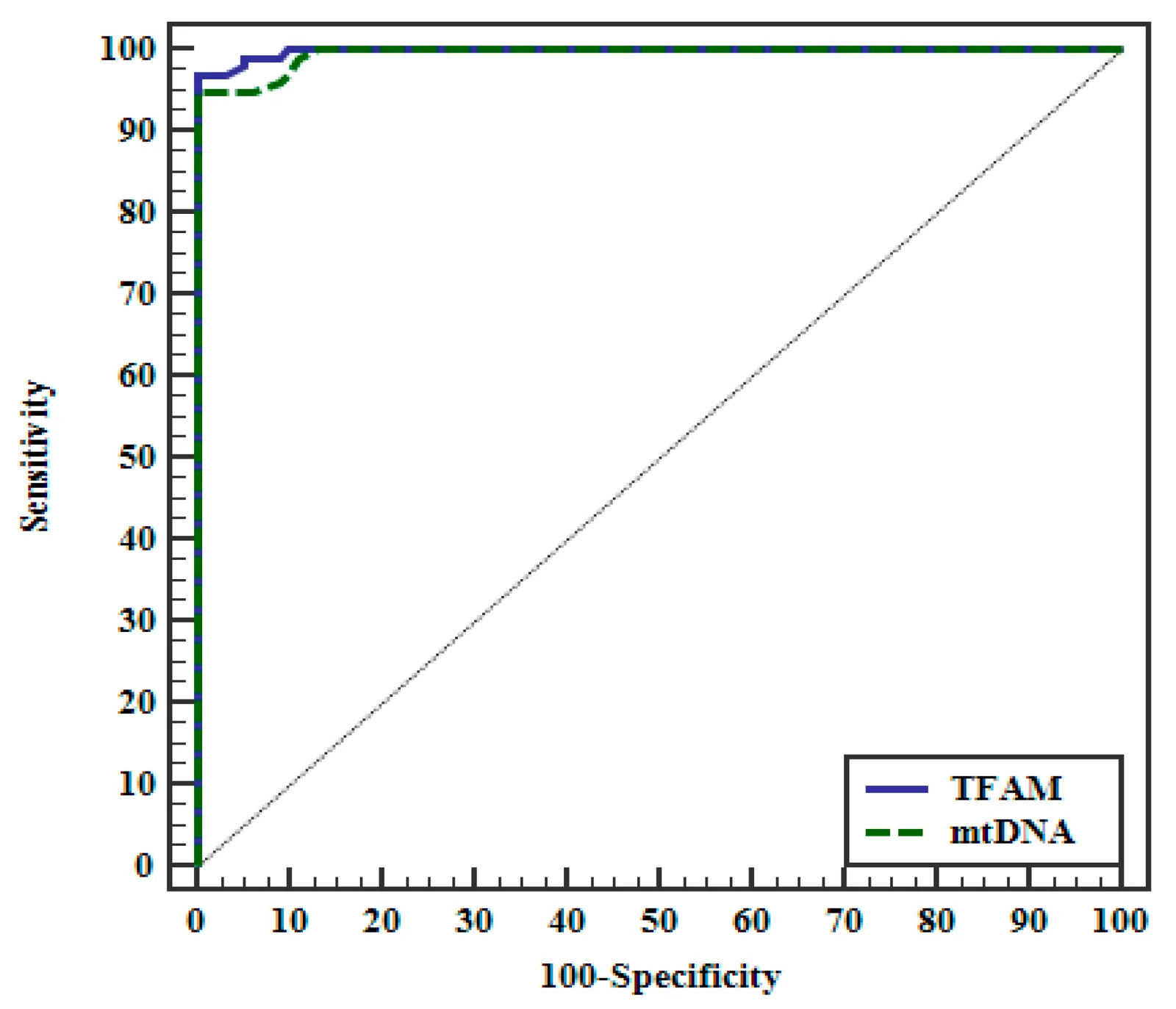
ROC curve for *TFAM* mRNA expression level and mtDNA CN to discriminate STEMI patients *n* = 100) from controls (*n* = 100).

Consistent with the very large effect size (Cohen’s d = −3.432), ROC analysis revealed excellent performance for *TFAM* mRNA expression being a biomarker associated with MI occurrence. At a cut-off ≤0.81, *TFAM* mRNA expression discriminated acute myocardial infarction patients from controls with AUC of 0.998 (95% CI: 0.995–1.0), sensitivity 98.0%, and specificity 95.0%. Similarly, mtDNA CN showed AUC of 0.995 (95% CI: 0.990–1.0), sensitivity 95.0%, and specificity 94.0% supporting the non-overlapping distribution observed (Table 4 and Figure 1). This performance has been done additionally after bootstrap. Supplementary ROC calculations used lower marker levels as evidence of MI. Apparent AUC intervals came from 2000 stratified bootstrap resamples, with random seed 20261007. Five-fold stratified cross-validation selected the Youden cutoff in each training fold and applied it to the held-out fold. *TFAM* mRNA expression training cutoffs were 0.79 in all folds; mtDNA cutoffs ranged from 0.74 to 0.77. Cross-validated sensitivity and specificity are aggregated held-out proportions, not externally validated estimates. The AUC shown remains the apparent rank-based AUC; it is not relabeled as a cross-validated model AUC (Supplementary Table S1).

**Figure 2:**
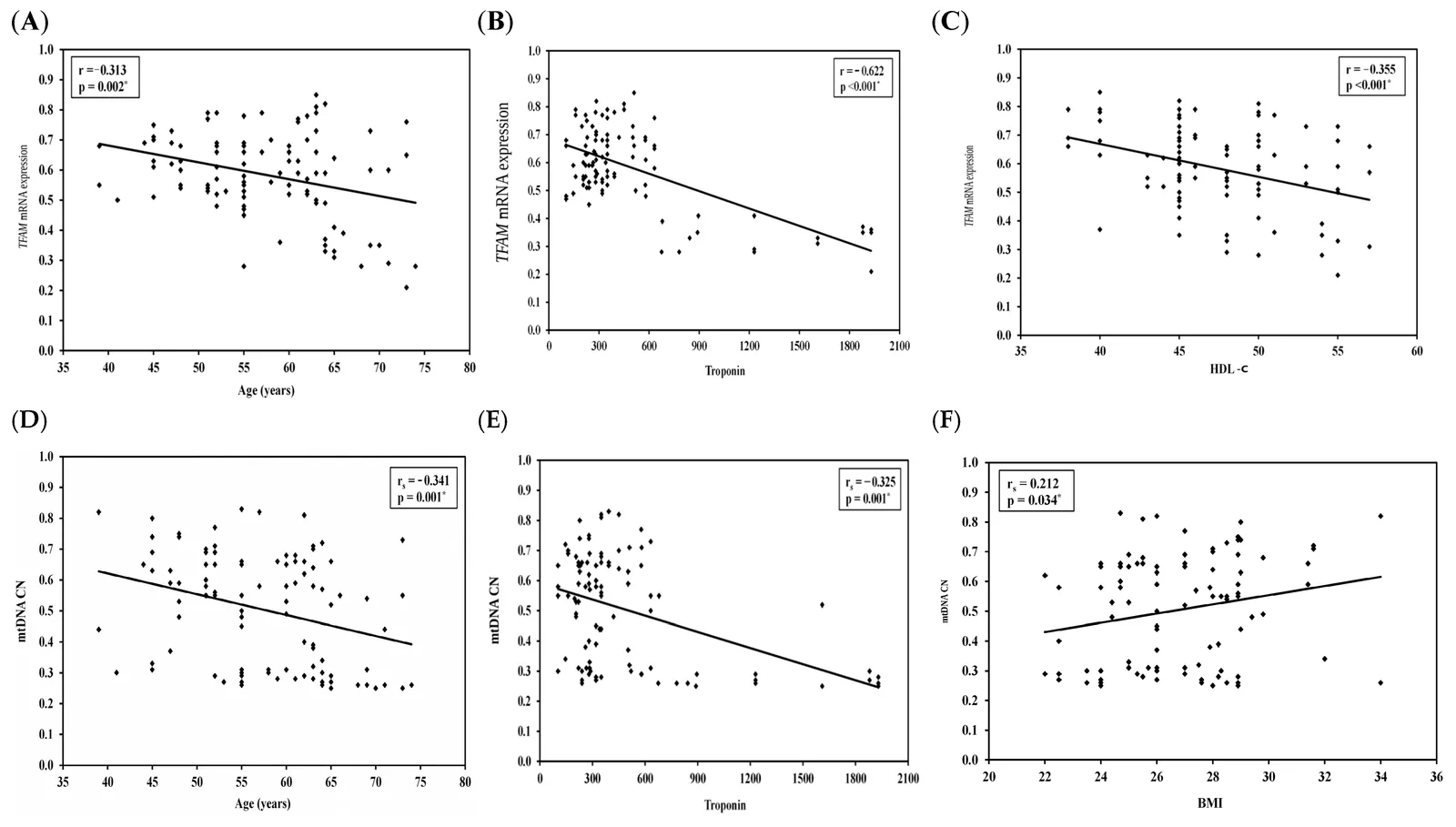
Correlation between *TFAM* gene expression and: (**A**) age; (**B**): troponin; (**C**): HDL-c in the patients’ group. Correlation between mitochondrial DNA copy number (mtDNA CN), and (**D**): age, (**E**): troponin, (**F**): BMI in the patients’ group (*n* = 100). HDL-c: high density lipoprotein cholesterol; BMI: body mass index; r: Pearson coefficient; rs: Spearman coefficient *: Statistically significant at *p* < 0.05.

We have also found some associations of great interest from correlation analysis. *TFAM* mRNA expression and mtDNA CN were markedly associated with age (*p* = 0.002 and 0.001, respectively) and troponin I levels (*p* < 0.001 and 0.001, respectively). One point was that there was a positive association between BMI and the number of mtDNA copies (*p* = 0.034). There was, however, a rather strong and negative association between TFAM mRNA level and the HDL-c level (*p* < 0.001). *TFAM* mRNA expression showed a statistically significant positive correlation with mtDNA CN (*p* < 0.001). After applying FDR correction separately for each set of correlations, all significant associations remained statistically significant except for the correlation between BMI and mtDNA CN, which lost statistical significance after FDR adjustment (Table 5, Figure 2). When age-adjusted partial correlations of *TFAM* mRNA level and mtDNA CN have been performed, all significant associations in Table 5 were still significant. In addition, CK-MB was significantly positively correlated with *TFAM* gene expression (*p* = 0.014), and triglycerides showed significant correlation with mtDNA CN (*p* = 0.029). After applying FDR correction separately for each set of age-adjusted partial correlations, all statistically significant associations remained significant except for the association between mtDNA CN and triglycerides, which became non-significant after FDR adjustment (Table 6).

A profound statistical preliminary relation was observed between rs1937 GC genotype and many factors like age, sex, and HDL-c showing large effect size (Cohen’s d = −1.430, 95% CI: −2.001 to −0.859, Phi = 0.285, 95% CI: 0.114–0.432 and Cohen’s d = −0.791, 95% CI: −1.335–−0.243 respectively) (Table 7). To determine the independent variables associated with myocardial infarction susceptibility, univariate and multivariate regression models were used. The multivariate regression analysis was adjusted by age, sex, BMI, smoking, and HDL-c. It showed that TFAM mRNA expression (OR = 0.421, 95% CI: 0.185–0.959, *p* = 0.039), mtDNA CN (OR = 0.464, 95% CI: 0.233–0.927, *p* = 0.030), and TFAM rs1937 GC genotype (OR = 9.475, 95% CI: 2.548–35.239, *p* = 0.001) were essential elements associated with MI occurrence (Table 8).

Firth penalized logistic regression was additionally performed to assess the robustness of the adjusted estimates in the presence of potential sparse-data instability. It showed that GC carriers had higher odds of MI than GG carriers (adjusted OR 6.263, 95% CI 1.965–23.887; *p* = 0.00147). The confidence interval remains wide. Higher *TFAM* mRNA expression and mtDNA copy number were associated with lower odds of MI; their near separation between groups produces very large estimated effects. These are sample associations, not protective causal effects (Table 9).

A Comparison between Firth penalized regression results and conventional maximum likelihood core models has been performed. The conventional models had finite fitted coefficients, but the marker estimates were much more extreme than the Firth estimates. Maximum likelihood estimate (*MLE*) intervals and *p* values use Wald inference, whereas Firth inference uses penalized profiles, so the *p* values are not directly interchangeable. Firth was chosen for the primary reporting because the sparse genotype counts and near separation make ordinary estimation sensitive. A small *p* value does not resolve sampling bias or establish clinical performance (Supplementary Table S2).

A model that included GC genotype, age, sex, BMI, smoking, hypertension, diabetes, LDL-c, HDL-c, and triglycerides (n = 200) has been done. The expanded genotype interval spans both lower and higher odds of MI; the data did not support describing GC as an independently confirmed MI risk factor after this adjustment, so it is better described to have a preliminary association with MI and should be studied in larger cohorts (Supplementary Table S3).

Patients carrying GC genotype of *TFAM* rs1937 variant showed a statistically significant lower mean ± SD *TFAM* mRNA expression level than the GG genotype 0.33 ± 0.05, *p* < 0.001 (Cohen’s d = 3.206, 95% CI: 2.503–3.898 representing a very large effect size) and, similarly, a lower mean ± SD mtDNA CN than the GG genotype 0.3 ± 0.09, *p* < 0.001 (r = 0.538, 95% CI: 0.382–0.664). No significant genotype-related differences were observed in the control group (*p* = 0.374 and 0.268, respectively), suggesting a disease-specific effect of the rs1937 variant on mitochondrial biogenesis under ischemic stress (Table 10).

## 3. Discussion

Myocardial infarction is a disorder in which blood flow to a particular part of the myocardium is either decreased or totally blocked. Myocytes, therefore, are deprived of oxygen, leading to mitochondrial alterations and eventually leading to the death of myocardial cells through necrosis [18,19]. Cardiac cells are densely packed with mitochondria to meet their high energetic demands. The involvement of these organelles in regulating oxidative stress at various steps can result in the survival or death of a cell [20]. Mitochondrial DNA CN reflects the capacity for mitochondrial DNA transcription and ATP production. This process is primarily regulated by TFAM, which binds to and coats mitochondrial DNA, thereby enhancing transcription and promoting genomic stability [21].

In this study, we attempted to estimate the role of mtDNA CN *TFAM* mRNA expression and its rs1937 variant in the pathophysiology of MI. Our results show that MI patients had a statistically significantly higher BMI than controls (*p* < 0.001), which aligns with findings by Wu et al., who reported that elevated BMI is associated with increased cardiovascular diseases [22].

This strengthens the interpretation that body weight affects MI risk via more than just increased workload and also through changes in metabolism and stress on the mitochondria. Campolo et al. demonstrated that obesity-driven mitochondrial dysfunction, specifically through oxidative damage and impaired mtDNA maintenance, is an essential pathway in earlier incidence of CAD, which reinforces the central role of mitochondrial mechanisms pinpointed in our study [23]. We found that MI patients were accompanied by dyslipidemia profile where LDL-c and total cholesterol were substantially high and HDL-c was reduced at the same time (*p* < 0.001), which coincides with the findings of Pietrangelo et al. who pinpointed the relationship between dyslipidemia and oxidative stress-induced mitochondrial dysfunction in cardiovascular disease [24]. Although serum levels of triglycerides showed no statistical difference (*p* = 0.085) between MI cases and controls, the trend toward elevation supports observations by Marino et al. who noted that triglyceride accumulation in obese individuals leads to lipotoxicity and impairs mitochondrial bioenergetics, particularly in adipose and cardiac tissues [25]. Obesity and dyslipidemia, therefore, are likely to synergistically trigger mitochondrial disturbance, which creates an underlying mechanism for the lowered *TFAM* mRNA expression and mtDNA levels seen in MI patients.

Elevated cardiac biomarkers, namely troponin I and CK-MB (*p* < 0.001) in MI cases, compared to controls are in line with mitochondrial dysfunction-related myocyte injury, as reported by Ruperez et al., who pinpointed mitochondrial oxidative stress as an essential factor that accelerates cardiovascular aging and vulnerability to ischemic damage [26]. The majority of the MI patients in our study had comorbidities of hypertension (50%) and diabetes mellitus (37%), which was in line with mitochondrial dysfunction in the context of cardiometabolic conditions that Pietrangelo et al. reported. This indicates that these diseases could cause vasoconstriction, lead to lower *TFAM* mRNA expression, and lower amount of mtDNA that cause cardiac muscle resistance impairment [24].

As presented in Table 2, the C allele and GC genotype of the *TFAM* rs1937 variant were profoundly more prevalent in MI patients compared to controls (*p* = 0.006 and 0.005, respectively). Our findings are partially consistent with those of Zheikova et al., who reported a significantly higher frequency of the rs1937 CC genotype among men with coronary artery disease compared with population controls [27]. In contrast, Palacín et al. found no significant association between *TFAM* polymorphisms and early-onset MI [28]. These discrepancies may reflect differences in genetic backgrounds, study populations, and sample sizes.

Further analysis demonstrated a statistically significant downregulation of both *TFAM* mRNA expression and mtDNA copy number in myocardial infarction patients relative to healthy controls (*p* < 0.001), representing biomarkers associated with MI susceptibility with AUC = 0.998 and 0.995, respectively. Also, we performed an additional sensitivity analysis restricted to participants without either hypertension or diabetes mellitus. This analysis included 36 MI patients and 100 controls. *TFAM* mRNA expression and mtDNA copy number remained markedly different between MI patients and controls (both *p* < 0.001). Kremer et al. demonstrated that reducing *TFAM* expression in mice induces tissue vulnerability and premature cardiac aging, which further supports that TFAM is an essential regulator of mitochondrial stability and cardiac resilience under stress [29]. Our findings are directly supported by Shaker (2024), who demonstrated that *TFAM* expression and mtDNA copy number were both profoundly suppressed in a rat model of isoproterenol-induced MI, which pinpoints their sensitivity to oxidative cardiac injury [30]. However, the very high AUCs for TFAM and mtDNA CN should be interpreted with caution, as they reflect an extreme case-control design comparing acute MI patients with super-healthy controls free of any comorbidities. This spectrum bias may inflate diagnostic performance compared to a real-world emergency setting, including patients with chest pain of non-cardiac origin. Future validation in an undifferentiated chest pain cohort is warranted.

The level of mitochondrial DNA in the blood could serve as a general inflammatory indicator in multiple diseases. The reduction of mtDNA and damage to the mitochondria are the consequence of the rise of oxidative stress and the decrease of the mitochondrial antioxidative capacity [31]. The results of the study by Schwarz et al. also confirmed that the low mtDNA copy number in humans was strongly linked to vascular calcification, an essential factor of atherosclerotic disease [32]. Reddy et al. also reported that reduction in the copy number of mtDNA may be an inherited risk factor for polycystic ovary syndrome [33]. Importantly, reduced mtDNA CN may not solely reflect an acute response to myocardial ischemia. Mitochondrial dysfunction, including alterations in mtDNA copy number and impaired mitochondrial dynamics (fusion/fission), has been increasingly recognized as a central mechanism in cardiovascular injury. Disruption of mitofusin-mediated fusion accelerates mitochondrial senescence and cardiac dysfunction [34], while correction of mitofusin conformations can restore mitochondrial function [35]. These findings support the biological plausibility that reduced mtDNA-CN, as a marker of mitochondrial compromise, is linked to cardiovascular injury. Prospective evidence has shown that lower leukocyte mtDNA-CN can precede cardiovascular events and is associated with subsequent incident coronary heart disease [36]. Moreover, reduced leukocyte mtDNA-CN has been reported during acute MI and was associated with subsequent adverse left ventricular remodeling [37]. These findings suggest that reduced mtDNA-CN may reflect an underlying mitochondrial phenotype, although additional modulation by the acute ischemic event cannot be excluded.

Our study revealed a profound inverse correlation between troponin levels and both *TFAM* mRNA expression and mtDNA CN, which implies a close link between the degree of mitochondrial dysfunction and the severity of myocardial injury. *TFAM* expression also negatively correlated with HDL-c levels (*p* < 0.001, and 0.002 when the correlation was adjusted by age). mtDNA CN showed a statistically substantial correlation with BMI (*p* = 0.034), but it lost the statistical significance after FDR adjustment. In the age-adjusted correlation model, the correlation between them was significant, *p* = 0.007, and it remained significant after FDR adjustment. In the age-adjusted partial correlation model, mtDNA CN showed a significant correlation with triglycerides (which became non-significant after FDR adjustment), while *TFAM* mRNA revealed a significant correlation with CK-MB. This research indicates that damaged mitochondria that show up in an acute MI could indicate the metabolic and vascular changes already going on in the body. Our results align with those of Shaker (2024), who reported a significant inverse relationship between mtDNA CN and cardiac biomarkers in rats with isoproterenol-induced MI, which supports the hypothesis that mitochondrial depletion parallels cardiomyocyte damage [30].

The study by Schwarz et al. also reported that there was a negative relationship between mtDNA CN and vascular calcification in humans, which pinpoints the role of mtDNA as an indicator of the total burden of cardiovascular conditions [32]. While no prior studies have directly reported a correlation between HDL-c levels and *TFAM* expression, this observed relationship in our cohort could suggest a previously underexplored link between lipid metabolism and mitochondrial biogenesis. This novel finding could reflect the influence of HDL-c on cellular oxidative balance and mitochondrial transcription, and it merits further mechanistic investigation.

A significant positive correlation was observed between *TFAM* mRNA expression and mtDNA copy number among STEMI patients (r = 0.541, *p* < 0.001), which remained significant after adjustment for age (partial r = 0.482, *p* < 0.001), and both results also remained significant after FDR correction for multiple tests. Consistent with this finding, Longo et al. [38]. reported a significant positive correlation between TFAM mRNA expression and mtDNA content in human preadipocytes (*p* = 0.011). Although the tissue type and clinical context differ from those of the present study, these findings provide supporting evidence for an association between TFAM expression and mtDNA content. Nevertheless, the observed correlation does not establish causality, and further studies are needed to clarify this relationship in acute myocardial infarction.

Analysis of the results in Table 7 showed that the GC genotype of the *TFAM* rs1937 polymorphism was linked to a substantially higher age and a greater likelihood of being male among STEMI patients when compared to the GG genotype (*p* < 0.001 and 0.004, respectively). Even though BMI, smoking, hypertension, and diabetes were not substantially different between the two genotypes, individuals with the GC had higher levels of troponin, as well as profound downregulation of *TFAM* and reduced levels of mtDNA CN (*p* < 0.001 for both). This indicates higher levels of mitochondrial dysfunction and cardiac injury severity. GC genotype generally tends to group with more advanced age, being male, and having less efficient mitochondrial indicators. This suggests that it could be a contributing factor genotype, which results in myocardial complications and related adverse outcomes to some extent [29,30].

The rs1937 variant is located within the mitochondrial targeting sequence of *TFAM* gene and may therefore influence mitochondrial targeting or processing of the TFAM protein. However, a direct effect of rs1937 on *TFAM* transcription has not been established. Interestingly, rs1937 has been reported to be linked with rs1049432, which was identified as a significant cis-eQTL for TFAM expression [39]. Moreover, the rs1049432 T allele has been associated with lower *TFAM* expression and is correlated with rs1937, suggesting that the observed association may partly reflect linked regulatory variation [40]. Thus, the observed association between rs1937 and TFAM expression may partly reflect linked regulatory variation rather than a direct transcriptional effect of rs1937. Given the essential role of TFAM in mtDNA maintenance and replication, alterations in TFAM availability may also contribute to changes in mtDNA copy number. Nevertheless, the causal relationship among these parameters remains uncertain and requires further functional investigations. These results generate the hypothesis that the C allele may act as a functional modifier associated with reduced *TFAM* expression and mtDNA content. However, a direct role in mitochondrial transcriptional repression was not demonstrated and remains to be functionally validated.

The present data further show a profound difference in HDL-c levels, with GC carriers having higher HDL-c (*p* = 0.005). This finding is counterintuitive and may reflect confounding, chance, or the small sample size of subgroups, particularly GC carriers. In the absence of longitudinal data and mechanistic experiments, we refrain from mechanistic interpretation and present this as an exploratory observation requiring replication.

Logistic regression analysis for factors affecting MI identified mitochondrial DNA CN, *TFAM* mRNA expression, and *TFAM rs1937* variant as essential biomarkers associated with myocardial infarction susceptibility. The univariate analysis revealed that reduced *TFAM* mRNA expression and lower mtDNA copy number were statistically significantly related to an elevated potential for MI susceptibility (*p* = 0.002 and 0.001, respectively). After accounting for major clinical variables as age, sex, BMI, smoking, and HDL-c, they were still statistically significant (*p* = 0.039 and 0.030, respectively). This result implies that decreased *TFAM* transcription might independently participate in myocardial vulnerability, likely through its central role in regulating mtDNA replication and mitochondrial function. The *TFAM* rs1937 GC genotype also had a strong independent association with MI susceptibility in the multivariable adjusted regression (adjusted OR = 9.457, *p* = 0.001). This suggests that this SNP could have functional implications in regulating transcriptional activity and mitochondrial biogenesis. These results are strongly supported by Seethashankar et al., who reviewed evidence linking mtDNA copy number depletion and TFAM dysregulation to various chronic diseases and emphasized their emerging role as biomarkers for mitochondrial and systemic dysfunction [41].

Table 10 illustrated that among STEMI patients, carriers of the GC genotype of the *TFAM* rs1937 variant exhibited substantially lower levels of both *TFAM* mRNA expression (mean ± SD: 0.33 ± 0.05) and mtDNA copy number (mean± SD: 0.30 ± 0.09) compared to GG genotype carriers (0.63 ± 0.10 and 0.54 ± 0.17, respectively) (*p* < 0.001). Yet no substantial differences were seen between the genotype groups in the control subjects. This indicates that ischemia or pathological stress increases the effect of the rs1937 C allele on mitochondrial function. These results are in line with earlier literature documenting a connection between the rs1937 C allele and an elevated risk of myocardial infarction, likely via impairing mitochondrial transcription efficiency and reducing mtDNA replication and overall mitochondrial integrity [28].

*TFAM* rs1937 variant was linked to lower mtDNA CN and oxidative stress in endometriosis [42,43]. This suggests that this genotype exerts systemic effects on mitochondrial homeostasis, which leads to mitochondrial dysfunction and diminished cellular energy capacity. This is consistent with the reduced *TFAM* expression observed in our study. Studies by Chen et al. also demonstrate that C allele carriers were less effective in terms of mitochondrial function and lived shorter. This indicates that mitochondrial and systemic longevity are affected by rs1937 [44]. This analysis therefore reinforces the notion that *TFAM* mRNA downregulation, *TFAM* rs1937 variant, and mtDNA copy number loss reflect mitochondrial instability and impaired biogenesis. This offers profound clinical value as promising non-invasive biomarkers associated with myocardial injury. This helps identify individuals at heightened MI risk and potential targets for early therapeutic intervention.

A limitation of this study is the absence of longitudinal pre-MI or post-recovery data; therefore, validation in larger independent cohorts and prospective longitudinal studies is warranted. An additional limitation is that *TFAM* mRNA expression and mtDNA copy number were measured in whole blood without adjustment for leukocyte subpopulations or platelet counts. Because blood-cell composition may change during acute MI and may influence whole-blood mitochondrial measurements, differences in cellular composition could have contributed to the observed associations, so further studies using cell-specific analyses and/or cardiac tissue, together with adjustment for leukocyte subsets and platelet counts, are required to determine the cellular origin and cardiac relevance of these findings.

An additional limitation is the case-control design with an artificially balanced proportion of myocardial infarction patients and controls (1:1), which does not reflect the actual prevalence of myocardial infarction in clinical populations. Consequently, the reported positive predictive value (PPV) and negative predictive value (NPV) are sample-specific and cannot be directly generalized to routine clinical practice because these measures depend on disease prevalence. Although AUC, sensitivity, and specificity are not directly determined by disease prevalence, they may be affected by the selected patient spectrum and case-control sampling. Therefore, prospective validation in representative clinical populations is required to establish the clinical applicability of these biomarkers. Also, a small number of C allele carriers of TFAM rs1937 variant and absence of CC genotype limited statistical power and requires replication in the larger Egyptian cohort.

## 4. Patients and Methods

### 4.1. Study Participants

This study was carried out as a collaborative effort between the Departments of Medical Biochemistry & Molecular Biology and Cardiology, Faculty of Medicine, Menoufia University. Two hundred participants were enrolled and divided into two groups.

Group 1 (STEMI Patients): This group included 100 patients diagnosed with myocardial infarction, recruited from the Coronary Care Unit (CCU) of Menoufia University Hospitals during the period from November 2024 until February 2025. The diagnosis of STEMI was made according to the latest guidelines using a combination of electrocardiographic elevation of the ST-segment in at least two consecutive leads, a clinical history of anginal discomfort, and high cardiac troponin levels [45].

Group 2 (Control Group): Controls were selected to be comparable with STEMI cases regarding age and sex. Controls were confirmed to be free from diabetes, hypertension, and any personal or family history of CAD through clinical examination and investigations. Demographic data of the participants were obtained, and body mass index (BMI) was determined by dividing weight by height squared (kg/m^2^). Exclusion Criteria: The study excluded individuals with congenital heart diseases, heart failure, any accompanying renal, hepatic, or autoimmune diseases. A participant flow diagram is illustrated in Supplementary Figure S1.

The STEMI patient presented to the hospital with varying time from the onset of the chest pain, with an average of 1 h from the pain onset and ranging from 30 min to 6 h. After patients were immediately diagnosed with STEMI, blood samples were withdrawn before starting medications. Coronary angiography (CAG) was performed using the Judkins technique guidelines [46]. Percutaneous Coronary Intervention (PCI) was performed for the culprit lesions as a reperfusion therapy. Coronary angiograms were digitally recorded for quantitative analysis.

In this study, most patients (50%) presented with anterior STEMI, and the culprit vessel was left anterior descending artery (LAD). Whereas 34%, 10%, 4% and 2% of STEMI patients presented with inferior, inferior posterior, lateral and inferior lateral STEMI respectively. A single dose of chewable aspirin, 300 mg, a loading dose of clopidogrel, 600 mg, or Ticagrelor, 180 mg, and a high-dose statin were administered on admission. Standard heparin 70 U/kg was also administered before the procedure. All primary PCI procedures were carried out by qualified interventional cardiologists via the femoral or radial arteries. In addition, 0.014-inch guidewires were used to pass the lesions. Based on coronary anatomy and lesion characteristics, patients underwent direct stenting, conventional stenting, or balloon dilation only. After the intervention, 75–150 mg/day of aspirin and 75 mg/day of clopidogrel or 90 mg BID of ticagrelor were administered.

### 4.2. Blood Sampling

Blood samples were collected from all participants immediately after diagnosis of MI and before beginning therapy. Each subject had a 7 mL venous blood sample taken and processed according to the following protocol: Two milliliters were put in an EDTA tube to extract the whole RNA to be used for measuring TFAM gene expression by real-time PCR; two more milliliters were added to another EDTA tube to extract DNA to detect mtDNA CN and TFAM rs1937 via the TaqMan allelic discrimination test method. Three more milliliters were collected into a plain tube (a tube with no anticoagulant). These samples were left to clot for 15 min before being put up for centrifugation at 4000 rpm for 10 min, resulting in serum separation. The serum was used for Laboratory investigations that included common colorimetric techniques utilizing kits from Spinreact (Girona, Spain) to measure serum levels of total cholesterol (TC) and triacylglycerol (TG) and a human kit (Wiesbaden, Germany) to measure serum HDL-c (high-density lipoprotein cholesterol). As TG values did not exceed 400 mg/dL, low-density lipoprotein cholesterol (LDL-c) concentration can be estimated using the formula (LDL-c) = TC − (TG/5 + HDL-c). Serum Troponin I and CK-MB were measured by Enzyme-Linked Fluorescent Assay (ELFA) technique using a miniVIDAS analyzer and VIDAS^®^ High-sensitive Troponin I and VIDAS CK-MB kits, respectively (BioMérieux SA, Mercy I’Etoile, France).

### 4.3. DNA Extraction

DNA was extracted from whole blood with the GENE JET Genomic DNA Extraction Kit (Cat. #K0781; Thermo Scientific, Vilnius, Lithuania) in accordance with the manufacturer’s guidelines, and its concentration, purity, and quality were verified using the N-60 Nanophotometer (Implen GmbH, Munich, Germany).

### 4.4. Genotyping of TFAM Polymorphisms (rs1937) by qPCR

**Figure 3:**
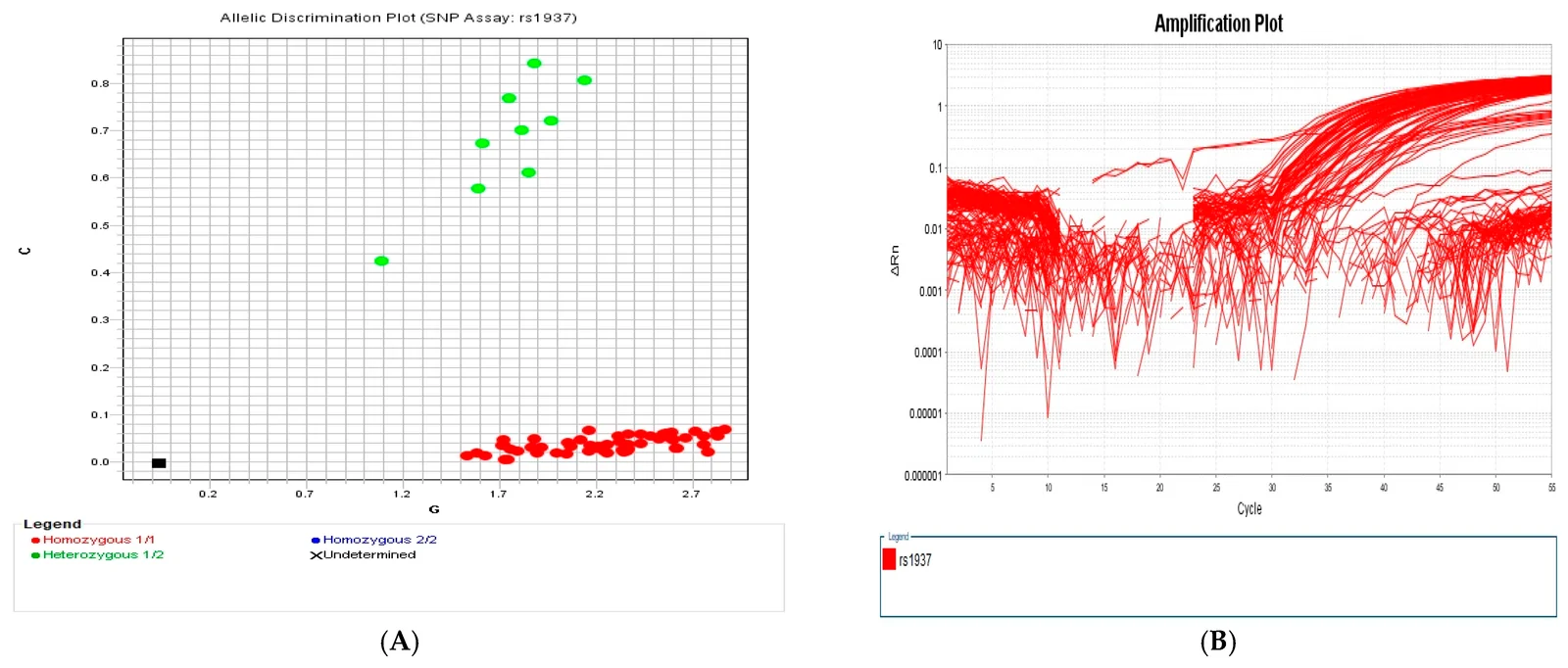
(**A**) Allelic discrimination plot of *TFAM* rs1937 variant. (**B**) The amplification plot of TFAM rs1937 variant.

The genotyping of rs1937 was carried out using real-time PCR for the allelic discrimination test. The TaqMan probe was utilized (Applied Biosystems, Foster City, CA, USA). Thermo Fisher Scientific supplied the primers, probes, and Genotyping Master Mix II (2x) (cat#.4371353). The sequence of the used probe was CTCCGAAGCATGTGGGGCGTGCTGA (C/G) TGCCCTGGGAAGGTCTGGAGCAGAG. Ten microliters of master mix, one microliter of probe/primer mix, and four microliters of DNase-free water were combined. For the negative control reaction, five microliters of DNase-free water were added, while five microliters of DNA extract were added to each test sample. The reactions were performed in the following sequence: first denaturation was carried out at 95 °C for 10 min, then 40 cycles of 15 s at 95 °C, and finally at 60 °C for 1 min. To ensure genotyping reproducibility, 10% of the DNA samples (randomly selected duplicate pairs) were re-genotyped in a blinded fashion, showing a 100% concordance rate. A QuantStudio 5 real-time PCR instrument (Applied Biosystems) running software version v1.5.2 was used to analyze the data (Figure 3). Furthermore, allele frequencies and genotype distributions for the *TFAM* rs1937 variant in global populations were retrieved from the NCBI dbSNP database (rs1937), including ALFA Project and the 1000 Genomes Project datasets and are presented in Supplementary Tables S4 and S5 [47,48].

### 4.5. Detection of mtDNA CN by qPCR

A portion of the mitochondrial genome was quantified using real-time PCR (qPCR). The amplification of a single-copy nuclear gene, glyceraldehyde 3-phosphate dehydrogenase (*GAPDH*), was used to normalize the mitochondrial target (*NADH* dehydrogenase 1, *ND1*) for relative measurement. The following primer sequences were used: for the *GAPDH* gene, sense 5′- GTCTCCTCTGACTTCAACAGCG -3′, antisense 5′- ACCACCCTGTTGCTGTAGCCAA -3′. The forward primer utilized for the mitochondrial ND1 gene was: 5′- GGGCTACTACAACCCTTCGCT -3′, while the reverse primer was 5′- GAGGCCTAGGTTGAGGTTGAC -3′. We prepared 20 µL qPCR reactions containing three microliters of DNA template, 10 microliters of Maxima SYBR Green qPCR Master Mix (2X) (Thermo Scientific, K0251), five microliters of RNase-free water, and one microliter of each forward and reverse primer. The settings for amplification were as follows: denaturation first for 10 min at 95 °C, then 40 cycles at 95 °C (15 s) and 60 °C (1 min). The threshold cycle (Ct) values for both the ND1 and GAPDH genes were recorded for each run. Every calculation was compared to a calibrator. In accordance with MIQE guidelines, the calibrator is a pooled genomic DNA sample prepared by mixing equal amounts of DNA from 10 healthy control participants. This calibrator was included in every qPCR run to control for inter-run variation [49].

**Figure 4:**
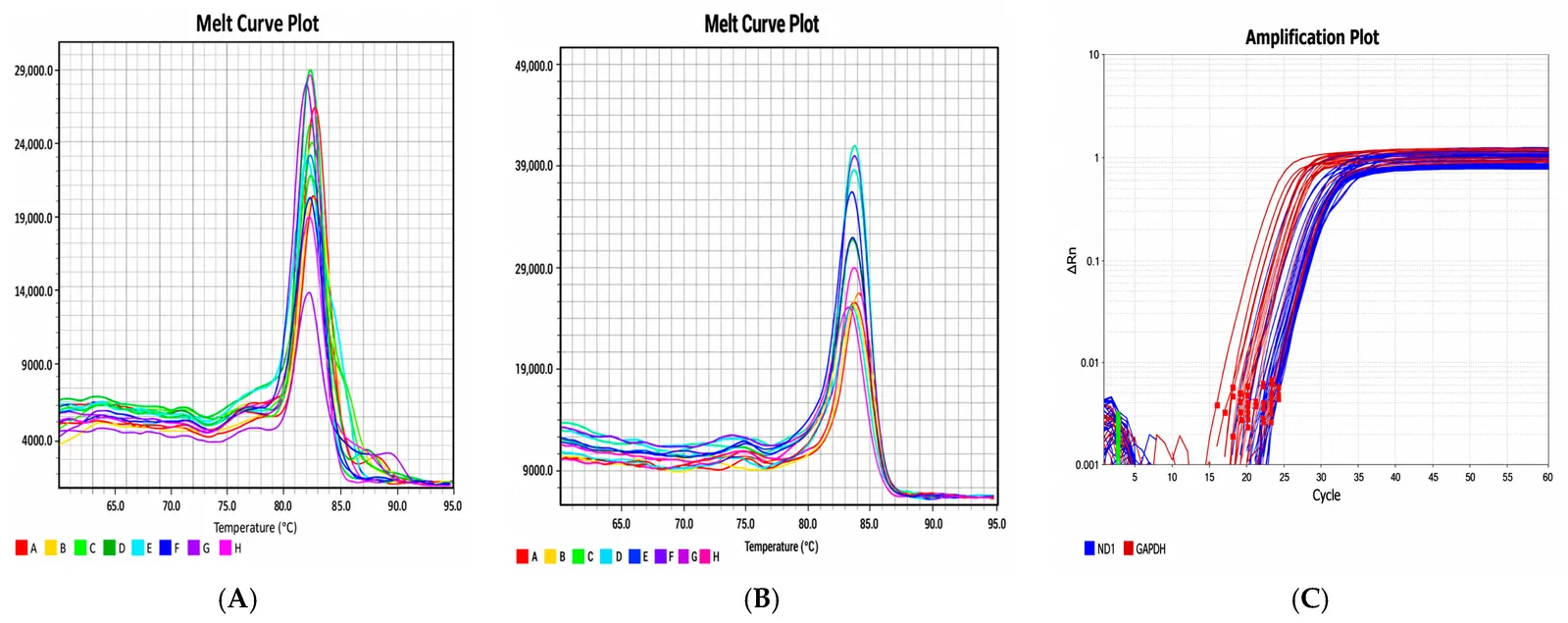
(**A**) Melting curve for ND1. (**B**) Melting curve for *GAPDH*. (**C**) Amplification plot for *ND1* and *GAPDH*.

The Ct value was computed as: relative mt DNA copy number = 2^−ΔΔCt^, where ΔCt is the CtND1 − CtGAPDH and ΔΔCt = ΔCt sample − ΔCt calibrator [50]. GAPDH was validated as a stable nuclear reference gene. Raw Ct values of GAPDH showed no significant difference between acute MI and control groups (*p* > 0.05) with low inter-sample variability. Data were processed using the Applied Biosystems QuantStudio 5 software (v1.5.2). Amplification specificity was verified, and primer-dimer artifacts were ruled out through melting curve analysis (Figure 4).

### 4.6. Detection of Mitochondrial Transcription Factor A Gene Expression (TFAM mRNA)

RNA was isolated from whole blood using kits from Qiagen, Hilden, Germany (QIAamp RNA Blood Mini Kits). In compliance with MIQE guidelines, RNA quality and purity were assessed by spectrophotometry (NanoPhotometer^®^ N60, Implen GmbH, Munich, Germany). RNA concentration was measured by detecting absorbance at 260 nm (A260). Absorbance readings (A260) should be greater than 0.15 to ensure significance. RNA purity was evaluated using the A260/A280 absorbance ratio, which indicates the level of protein contamination. Pure RNA typically has a ratio between 1.8 and 2.1. A260/A280 ratio greater than 1.6 was accepted. Samples with ratios below 1.5 were considered insufficiently pure and required re-extraction.

To prevent RNA degradation, all procedures were performed on ice using RNase-free and DNase-free pipet tips, with gloves and RNase decontamination solution. All steps were performed in laminar flow hood to avoid cross-contamination. The extract was stored at −80 °C.

cDNA synthesis was performed using the RevertAid First Strand cDNA Synthesis kit (Thermo Fisher Scientific, Vilnius, Lithuania). A fixed input of 150 ng of total RNA was used. The reaction was chilled on ice and consisted of reaction buffer (4 μL), nuclease-free H2O (3 μL), dnTPMix (2 μL), extracted RNA (8 μL), RevertAid RT (1 μL) (200 U/mL), Ribolock RNase inhibitor (1 μL), and random primer hexamer (1 μL), making up the volume of 20 microliters. The Biometra thermal cycler (Biometra GmbH, Göttingen, Germany) was used. was used and adjusted for 25 °C for 5 min, 60 min at 42 °C, then 5 min at 70 °C. For the PCR, the resultant cDNA was kept at −20 °C. cDNA amplification was detected by Master Mix of Maxima SYBR Green (Vilnius, Lithuania, K0251, Thermo Scientific) for quantitative real-time PCR using the designed primers supplied by (Invitrogen, Thermo Fisher Scientific, Carlsbad, CA, USA). The forward primer for the *TFAM* gene (XM_011540121.4) is 5′-ATGGCGTTTCTCCGAAGCAT-3′ and the reverse primer is 5′-TCCGCCCTATAAGCATCTTGA-3′. The forward and reverse primers for the reference gene *GAPDH* were 5′-CCACTCCTCCACCTTTGAC-3′, and 5′-ACCCTGTTGCTGTAGCCA-3′.

In addition, 20 µL total volume of qPCR samples consisted of SYBR green master mix (10 μL), forward primer (1 μL), reverse primer (1 μL), nuclease-free H2O (7 μL), and cDNA product (1 μL). Data were analyzed using QuantStudio 5 (Applied Biosystems v1.5.2. The program of cycling was as follows: 10 min of denaturation initially at 95 °C that were followed by 40 cycles of denaturation at 95 °C (15 s) and 1 min of annealing and extension at 60 °C. For each qPCR run, a no-template control (RNase-free water instead of cDNA) and a no-reverse-transcription control (RNA sample processed without reverse transcriptase enzyme to control for genomic DNA contamination) were included. No amplification was observed in either control, confirming absence of reagent contamination and genomic DNA. To minimize inter-run variation, all qPCR runs were performed using the same threshold setting (0.1) and baseline. An inter-run calibrator was included in each plate in triplicate. Patient and control samples were randomized across plates to avoid batch effect. Also, 10% of samples were run in duplicate. All samples had difference in Ct standard deviation <0.5 between replicates. No outliers were detected, and therefore no samples were excluded.

**Figure 5:**
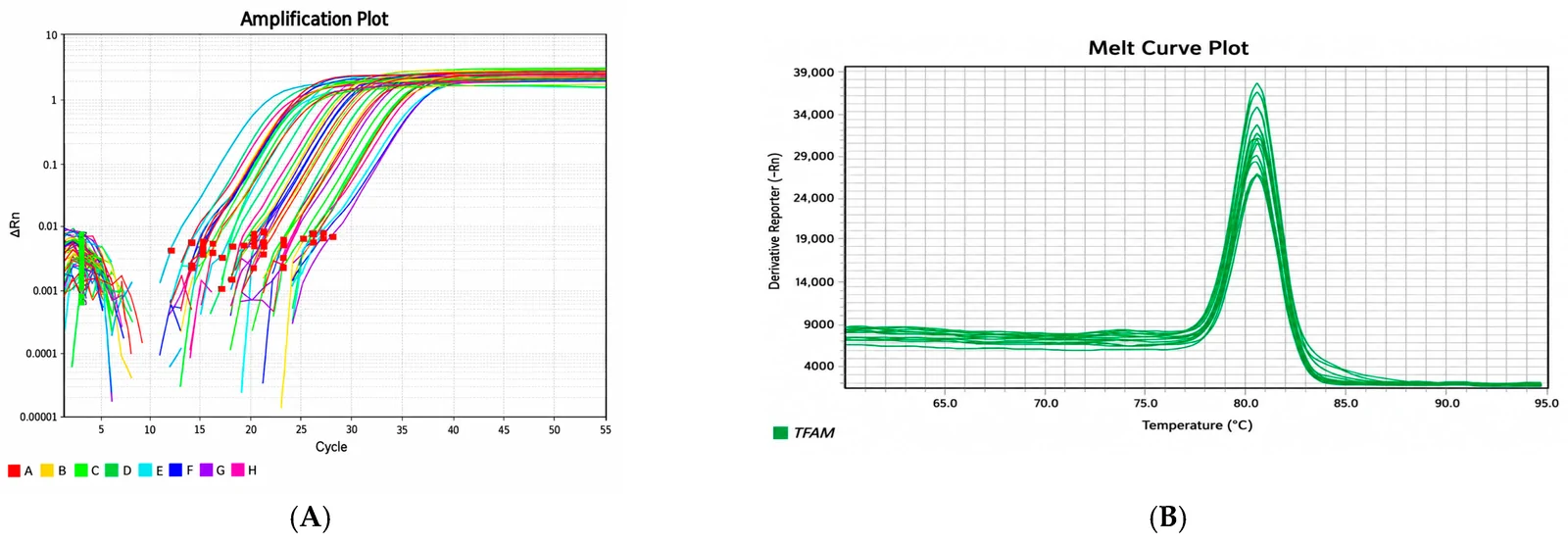
(**A**) Amplification plot for *TFAM* mRNA expression. (**B**) Melting curve for *TFAM* mRNA.

Relative detection of gene expression of *TFAM* was accomplished using the 2^−ΔΔCt^ comparative method, assuming ~100% amplification efficiency for both target and reference genes based on validated manufacturer primers and on the previous literature. Specificity was confirmed by melt curve analysis showing a single peak for each gene. The melting curve analysis was used to validate the identity and specificity of PCR products and prevention of primer dimers (Figure 5).

### 4.7. Statistical Analysis of the Data

IBM SPSS Statistics for Windows, Version 20.0 (IBM Corp., Armonk, NY, USA) was used to conduct statistical analyses). Firth bias-reduced logistic regression was conducted using the logistf package in R. A post-hoc power analysis was performed for *TFAM* mRNA expression levels using G*Power software (version 3.1.9.4). The analysis was based on a two-independent-samples *t*-test with a two-tailed significance level (α) of 0.05 and a total sample size of 200 participants (100 myocardial infarction patients and 100 healthy controls). Based on the observed mean values and standard deviations (0.58 ± 0.15 for patients vs. 1.04 ± 0.12 for controls), the calculated Cohen’s d effect size was 3.38. The resulting statistical power (1–β) was 0.999 (99.9%), demonstrating that the sample size was fully adequate to detect statistically significant differences between the studied groups [51].

Categorical variables were summarized as frequencies and percentages and compared using the Chi-square test or Fisher’s exact test, as appropriate. The normality of continuous variables was assessed using the Shapiro–Wilk test. For instances where over 20% of cells in a contingency table had an expected count below 5, the Monte Carlo correction was applied. The Kolmogorov-Smirnov test was used to assess the normality of quantitative variables. Normally distributed continuous data are expressed as mean ± standard deviation along with the median. The two groups were compared using the Student’s t-test. The Mann-Whitney U test was used to compare non-normally distributed data. Effect sizes were reported using Cohen’s d for parametric comparisons, the rank-based effect size (r) for Mann–Whitney U tests, and the phi coefficient (φ) for categorical associations, with corresponding 95% confidence intervals (CIs) where available. Pearson’s or Spearman’s correlation coefficient was used to assess associations between quantitative variables, according to their distribution. Age-adjusted partial correlation analysis was additionally performed to assess the relationships between TFAM mRNA expression, mitochondrial DNA copy number (mtDNA CN), and clinical parameters while controlling for age.

Hardy–Weinberg equilibrium was assessed using the Chi-square goodness-of-fit test. Univariable and multivariable binary logistic regression analyses were performed to evaluate the associations of *TFAM* mRNA expression, mtDNA CN, and TFAM rs1937 genotype with myocardial infarction status. Separate adjusted regression models were constructed for the investigated parameters, controlling for age, sex, body mass index (BMI), smoking status, and high-density lipoprotein cholesterol (HDL-c). Covariates were selected according to their clinical relevance and potential confounding effects. Multicollinearity was evaluated using variance inflation factors (VIFs) and correlation matrices, while conventional logistic regression model fit was assessed using the Hosmer–Lemeshow goodness-of-fit test.

An additional sensitivity analysis was conducted to address potential confounding associated with hypertension and diabetes mellitus. To control for multiple hypothesis testing, *p*-values were adjusted using the False Discovery Rate (FDR) according to the Benjamini–Hochberg procedure, with adjusted q-values < 0.05 considered statistically significant. Utilizing logistic regression, data were analyzed to determine which independent factor has the most significant effect on MI. Firth penalized logistic regression was additionally performed to assess the robustness of the adjusted estimates in the presence of potential sparse-data instability [52,53,54]. Tests were deemed statistically significant if the *p*-value was less than 0.05.

## 5. Conclusions

This study established that reduced mtDNA copy number and decreased *TFAM* mRNA expression are significant biomarkers associated with acute myocardial infarction in the Egyptian population, underscoring their crucial function in disease development. The GC genotype and C allele of *TFAM* rs1937 variant showed a preliminary association with MI in STEMI patients. To completely clarify how MI, *TFAM* mRNA expression, and mtDNA CN are related mechanistically, future longitudinal studies with larger cohorts are warranted. Nonetheless, these findings provide a foundational framework for advanced statistical analyses, functional investigations, and the potential development of potential biomarkers and therapeutic strategies.

## Figures and Tables

**Table 1:** Comparison of Patient and Control Groups by Clinical and Demographic Parameters.

	Patients (*n* = 100)	Controls (*n* = 100)	Test of Sig.	*p*	Effect Size (95% CI)
**Age (Years)**					
Mean ± SD.	57.40 ± 8.12	55.36 ± 8.66	t = 1.718	0.087	Cohen’s d = 0.243 (−0.035–0.521)
Median (IQR)	58.0 (52.0–63.0)	55.0 (48.0–62.0)			
**Sex (No/%)**					
Male	55 (55.0%)	68 (68.0%)	χ^2^ = 3.569	0.059	Phi = 0.134 (−0.016–0.265)
Female	45 (45.0%)	32 (32.0%)			
**BMI (kg/m^2^)**					
Mean ± SD.	26.76 ± 2.54	25.32 ± 1.60	t = 4.781 *	<0.001 *	Cohen’s d = 0.676 (0.390–0.960)
Median (IQR)	27.0 (24.85–28.50)	25.0 (24.0–26.25)			
**Smoking (No/%)**	37 (37.0)	25 (25.0%)	χ^2^ = 3.366	0.067	Phi = 0.130 (−0.028–0.267)
**Hypertension (No/%)**	50 (50.0%)	0 (0.0%)	χ^2^ = 66.667 *	<0.001 *	Phi = 0.577 (−0.503–0.658)
**DM (No/%)**	37 (37.0)	0 (0.0%)	χ^2^ = 45.399 *	<0.001 *	Phi = 0.476 (0.399–0.556)
**Troponin I (ng/L)**					
Mean ± SD.	485.76 ± 434.21	11.35 ± 4.59	U = 0.0 *	<0.001 *	r = 0.864 (0.824–0.895)
Median (IQR)	320 (240–549)	10.50 (7.12–16.35)			
**CK-MB (ng/mL)**					
Mean ± SD.	25.51 ± 50.80	3.12 ± 0.99	U = 309.0 *	<0.001 *	r = 0.811 (0.758–0.854)
Median (IQR)	12.0 (6.90–18.50)	3.40 (1.99–3.90)			
**Total cholesterol (mg/dL)**					
Mean ± SD.	202.9 ± 41.36	154.8 ± 10.48	t = 11.259 *	<0.001 *	Cohen’s d = 1.592 (1.272–1.909)
Median (IQR)	195.0 (176.50–230.0)	154.5 (148.0–160.50)			
**LDL-c (mg/dL)**					
Mean ± SD.	122.1 ± 36.78	84.74 ± 10.99	t = 9.739 *	<0.001 *	Cohen’s d = 1.377 (1.067–1.684)
Median (IQR)	121.0 (98.0–144.0)	84.50 (79.0–89.0)			
**HDL-c (mg/dL)**					
Mean ± SD.	47.38 ± 4.48	49.59 ± 5.37	t = 3.160 *	0.002 *	Cohen’s d = −0.447 (−0.728 to −0.166).
Median (IQR)	46.0 (45.0–50.0)	49.0 (46.0–54.0)			
**Triglycrides (mg/dL)**					
Mean ± SD.	137.0 ± 35.77	132.3 ± 11.85	U = 4925.0	0.854	r = 0.018 (−0.179–0.214)
Median (IQR)	130.0 (110.0–153.0)	130.0 (124.0–145.0)			
***TFAM* mRNA level**					
Mean ± SD.	0.58 ± 0.15	1.04 ± 0.12	t = 24.270 *	<0.001 *	Cohen’s d = −3.432 (−3.867–−2.993)
Median (IQR)	0.60 (0.51–0.69)	1.04 (0.97–1.15)			
**mtDNA CN**					
Mean ± SD.	0.50 ± 0.18	1.10 ± 0.17	U = 50 *	<0.001 *	r = 0.857 (0.815–0.890)
Median (IQR)	0.55 (0.30–0.66)	1.10 (0.93–1.21)			

IQR: Interquartile range; LDL-c: low density lipoprotein cholesterol; HDL-c: high density lipoprotein cholesterol; BMI: body mass index; CK-MB: creatine kinase-MB; mtDNA CN: mitochondrial DNA copy number; SD: Standard deviation; χ^2^: Chi-square test; U: Mann-Whitney test; t: Student *t*-test; CI: confidence interval; p: *p*-value for comparing between the two studied groups; *: Statistically significant at *p* < 0.05. After applying the Benjamini–Hochberg false discovery rate (FDR) correction, all statistically significant associations remained significant.

**Table 2:** Comparison between STEMI patients and controls according to *TFAM* rs1937 variant.

	Patients (*n* = 100)	Controls (*n* = 100)	χ^2^	P2	OR (LL–UL 95% CI)	P3
**rs1937**						
GG	84 (84.0%)	96 (96.0%)	8.0 *	0.005 *	1.000	
GC	16 (16.0%)	4 (4.0%)			4.571 (1.471–14.210)	0.009 *
CC	0 (0.0%)	0 (0.0%)			–	–
**^HW^** **p_1_**	0.385	0.838				
**Allele**						
G	184 (92.0%)	196 (98.0%)	7.579 *	0.006 *		
C	16 (8.0%)	4 (2.0%)			4.261 (1.399–12.981)	0.011 *

χ^2^: Chi-square test; LL: Lower limit; UL: Upper Limit; CI: Confidence interval; OR: odds ratio; HWp1: *p* value for Chi-square for goodness of fit for Hardy-Weinberg equilibrium; P2: *p* value to compare between the two studied groups; p3: *p* value for odds ratio to compare between the two studied groups; *: Statistically significant at *p* < 0.05. After applying FDR correction, all statistically significant associations remained significant.

**Table 3:** Sensitivity analysis comparing STEMI patients without hypertension or diabetes mellitus with controls.

	Patients Without HTN and DM (*n* = 36)	Controls (*n* = 100)	Test of Sig.	*p*	Effect size (95% CI)
***TFAM* mRNA level**					
Mean ± SD.	0.59 ± 0.12	1.04 ± 0.12	t = 19.028 *	<0.001 *	Cohen’s d = 3.698 (3.111–4.279)
Median (IQR)	0.60 (0.53–0.69)	1.04 (0.97–1.15)			
**mtDNA CN**					
Mean ± SD.	0.52 ± 0.16	1.10 ± 0.17	U = 9.50 *	<0.001 *	r = 0.761 (0.679–0.824)
Median (IQR)	0.56 (0.34–0.65)	1.10 (0.93–1.21)			
**rs1937**					
GG	33 (91.7%)	96 (96.0%)	FE	0.381	Phi = 0.087 (−0.110–0.265)
GC	3 (8.3%)	4 (4.0%)			
CC	0 (0.0%)	0 (0.0%)			

IQR: Inter quartile range; SD: Standard deviation; FE: Fisher’s Exact test; CI: confidence interval; U: Mann Whitney test; t: Student *t*-test; HTN: hypertension; DM: diabetes mellitus; *p*: *p* value for comparing between the two studied groups; *: Statistically significant at *p* < 0.05.

**Table 4:** Discriminative performance for *TFAM* mRNA expression level and mtDNA CN for differentiating STEMI patients (*n* = 100) from controls (*n* = 100).

	AUC	*p*	95% CI	Cut Off	Sensitivity	Specificity	PPV	NPV
***TFAM* mRNA expression level**	0.998	<0.001 *	0.995–1.0	≤0.81	98.0	95.0	95.1	97.9
**mtDNA CN**	0.995	<0.001 *	0.990–1.0	≤0.79	95.0	94.0	94.1	94.9

AUC: Area Under the Curve value; Probability value; CI: Confidence Interval; PPV: Positive predictive value; NPV: Negative predictive value *: Statistically significant at *p* < 0.05. PPV and NPV were calculated from the study’s balanced case-control sample (50% cases and 50% controls) and are therefore sample-specific estimates that should not be directly extrapolated to populations with different myocardial infarction prevalences.

**Table 5:** Correlation between *TFAM* mRNA expression level and mtDNA CN with clinical and demographic parameters in patient group (*n* = 100).

	TFAM mRNA Level		mtDNA CN	
	r	*p*	r_s_	*p*
**Age**	−0.313	0.002 *	−0.341	0.001 *
**HR**	−0.019	0.851	−0.069	0.495
**SBP**	−0.009	0.926	−0.096	0.343
**DBP**	0.036	0.721	−0.009	0.927
**Troponin I**	−0.622	<0.001 *	−0.325	0.001 *
**CK-MB**	0.193	0.054	0.170	0.092
**Total cholesterol**	0.041	0.684	0.066	0.516
**LDL-c**	0.039	0.697	0.092	0.365
**BMI**	0.072	0.477	0.212	0.034 *
**HDL-c**	−0.355	<0.001 *	−0.158	0.116
**Triglycrides**	0.122	0.227	0.149	0.138
**mtDNA CN**	0.541	<0.001 *	-	-

HR: heart rate; SBP: systolic blood pressure; DBP: diastolic blood pressure; LDL-c: low density lipoprotein cholesterol; HDL-c: high density lipoprotein cholesterol; BMI: body mass index; CK-MB: creatine kinase-MB; mtDNA CN: mitochondrial DNA copy number; r: Pearson coefficient; rs: Spearman coefficient *: Statistically significant at *p* < 0.05. After applying FDR correction separately for each set of correlations, all significant associations remained statistically significant except for the correlation between BMI and mtDNA CN, which lost statistical significance after FDR adjustment.

**Table 6:** Age-adjusted partial correlations of *TFAM* mRNA expression level and mtDNA CN with clinical and demographic parameters among MI patients (STEMI patients) (*n* = 100).

	TFAM mRNA Level		mtDNA CN	
	Partial r	*p*	Partial r	*p*
**HR**	−0.160	0.115	−0.194	0.054
**SBP**	0.094	0.356	0.008	0.941
**DBP**	0.145	0.153	0.091	0.372
**Troponin I**	−0.572	<0.001 *	−0.353	<0.001 *
**CK-MB**	0.246	0.014 *	0.084	0.410
**Total cholesterol**	0.042	0.677	0.137	0.176
**LDL-c**	0.028	0.785	0.145	0.152
**BMI**	0.121	0.233	0.270	0.007 *
**HDL-c**	−0.303	0.002 *	−0.099	0.328
**Triglycrides**	0.138	0.172	0.220	0.029 *
mtDNA CN	0.482	<0.001 *	-	-

HR: heart rate; SBP: systolic blood pressure; DBP: diastolic blood pressure; LDL-c: low density lipoprotein cholesterol; HDL-c: high density lipoprotein cholesterol; BMI: body mass index; CK-MB: creatine kinase-MB; mtDNA CN: mitochondrial DNA copy number Partial r: Partial correlation coefficient adjusted for age; *: Statistically significant at *p* < 0.05. After applying FDR correction separately for each set of age-adjusted partial correlations, all statistically significant associations remained significant except for the association between mtDNA CN and triglycerides, which became non-significant after FDR adjustment.

**Table 7:** Relation between *TFAM* rs1937 variant and both clinical and demographic parameters in STEMI patients (*n* = 100).

	rs1937		Test of Sig.	*p*	Effect Size (95% CI)
	GG (n = 84)	GC (n = 16)			
**Age (Years)**					
Mean ± SD.	55.75 ± 7.56	66.06 ± 4.85	t = 7.031 *	<0.001 *	Cohen’s d = −1.430 (−2.001 to −0.859)
Median (IQR)	55.0 (51.0–62.0)	65.0 (63.0–68.50)			
**Sex (No/%)**					
Male	41 (48.8%)	14 (87.5%)	χ^2^ = 8.129 *	0.004 *	Phi = 0.285 (0.114–0.432)
Female	43 (51.2%)	2 (12.5%)			
**BMI (kg/m^2^)**					
Mean ± SD.	26.79 ± 2.42	26.57 ± 3.15	t = 0.324	0.747	Cohen’s d = 0.088 (−0.447–0.623)
Median (IQR)	26.50 (25.0–28.50)	27.30 (24.0–28.70)			
**Smoking (No/%)**	32 (38.1%)	5 (31.3%)	χ^2^ = 0.270	0.603	Phi = 0.052 (−0.148–0.229)
**Hypertension (No/%)**	40 (47.6%)	10 (62.5%)	χ^2^ = 1.190	0.275	Phi = 0.108 (−0.084–0.276)
**DM (No/%)**	33 (39.3%)	4 (25.0%)	χ^2^ = 1.177	0.278	Phi = 0.108 (−0.097–0.276)
**Troponin I (ng/L)**					
Mean ± SD.	325.6 ± 137.0	1326.4 ± 493.1	U = 0.0	<0.001 *	r = 0.633 (0.498–0.737)
Median (IQR)	283.5 (227.0–392.0)	1230.0 (866.5–1880.0)			
**CK-MB (ng/mL)**					
Mean ± SD.	28.05 ± 55.02	12.19 ± 7.49	U = 540.50	0.215	r = 0.124 (−0.074–0.313)
Median (IQR)	12.0 (9.60–19.20)	12.20 (5.75–15.0)			
**Total cholesterol (mg/dL)**					
Mean ± SD.	202.5 ± 41.49	204.9 ± 41.96	t = 0.219	0.827	Cohen’s d = 0.060 (−0.475–0.594)
Median (IQR)	195.0 (178.50–220.0)	213.0 (165.50–238.0)			
**LDL-c (mg/dL)**					
Mean ± SD.	121.7 ± 36.44	124.5 ± 39.61	t = 0.281	0.779	Cohen’s d = 0.077 (−0.458–0.611)
Median (IQR)	121.0 (99.0–133.0)	130.0 (86.50–163.0)			
**HDL-c (mg/dL)**					
Mean ± SD.	46.83 ± 4.28	50.25 ± 4.54	t = 2.901 *	0.005 *	Cohen’s d = −0.791 (−1.335–−0.243)
Median (IQR)	45.0 (45.0–50.0)	50.0 (48.0–54.0)			
**Triglycerides (mg/dL)**					
Mean ± SD.	139.0 ± 37.51	126.6 ± 22.93	U = 559.0	0.287	r = 0.106 (−0.092–0.297)
Median (IQR)	130.0 (110.50–155.50)	120.0 (110.0–148.50)			

LDL-c: low density lipoprotein cholesterol; HDL-c: high density lipoprotein cholesterol; BMI: body mass index; CK-MB: creatine kinase-MB; IQR: Interquartile range; t: Student *t*-test; χ^2^: Chi square test; U: Mann-Whitney test; MC: Monte Carlo test; SD: Standard deviation; CI: confidence interval; *p*: *p*-value for comparing between the two studied groups *: Statistically significant at *p* < 0.05. After applying the Benjamini–Hochberg false discovery rate (FDR) correction, all statistically significant associations remained significant.

**Table 8:** Univariate and multivariate logistic regression analysis for factors affecting myocardial infarction.

	Univariate		Multivariable	
	*p*	COR (95% CI)	*p*	AOR (95% CI)
***TFAM* mRNA expression ^&^**	0.002 *	0.514 (0.334–0.791)	0.039 *	0.421 (0.185–0.959)
**mtDNA CN ^&^**	0.001 *	0.643 (0.500–0.828)	0.030 *	0.464 (0.233–0.927)
**TFAM rs1937**				
**GG (ref)**		1.000		
**GC**	0.009 *	4.571 (1.471–14.210)	0.001 *	9.475 (2.548–35.239)

CI: Confidence interval; COR: crude odds ratio; AOR: adjusted odds ratio; adjusted for age, sex, BMI, smoking, and HDL-c *: Statistically significant at *p* < 0.05 ^&^: TFAM mRNA and mtDNA CN values were multiplied by 100 before inclusion in the logistic regression model; therefore, the reported ORs correspond to a 0.01-unit increase in the original marker value.

**Table 9:** Crude and core adjusted Firth associations with STEMI.

Exposure	Crude OR (95% CI)	Core Adjusted OR (95% CI)	*p*	*FDR p*
**GC**	4.187 (1.528 to 13.992)	6.263 (1.965 to 23.887)	0.001 *	0.001 *
***TFAM*** ** mRNA** **expression per 0.1 unit**	0.004 (3.13 × 10^−5^ to 0.058)	0.013 (7.25 × 10^−5^ to 0.098)	<0.001 *	<0.001 *
**mtDNA CN** **per 0.1 unit**	0.024 (0.001 to 0.112)	0.036 (3.10 × 10^−4^ to 0.152)	<0.001 *	<0.001 *

CI: Confidence interval; OR: odds ratio; AOR: adjusted odds ratio; FDR: false discovery rate correction; mtDNA CN: mitochondrial DNA copy number; *: Statistically significant at *p* < 0.05.

**Table 10:** Relation between *TFAM* rs1937 variant and *TFAM* mRNA expression levels and mtDNA CN in each group.

		rs1937		Test of Sig.	*p*	Effect Size (95% CI)
		GG	GC			
***TFAM* mRNA expression**	**Patient**	**(*n* = 84)**	**(*n* = 16)**			
	Mean ± SD.	0.63 ± 0.10	0.33 ± 0.05	t = 17.354 *	<0.001 *	Cohen’s d = 3.206 (2.503–3.898)
	Median (IQR)	0.63 (0.55–0.70)	0.34 (0.28–0.37)			
	**Control**	**(*n* = 96)**	**(*n* = 4)**			
	Mean ± SD.	1.04 ± 0.12	1.09 ± 0.16	t = 0.893	0.374	Cohen’s d = 0.456 (−0.547–1.457)
	Median (IQR)	1.04 (0.96–1.15)	1.15 (1.0–1.18)			
**mtDNA CN**	**Patient**	**(*n* = 84)**	**(*n* = 16)**			
	Mean ± SD.	0.54 ± 0.17	0.30 ± 0.09	U = 100.50 *	<0.001 *	r = 0.538 (0.382–0.664)
	Median (IQR)	0.58 (0.37–0.66)	0.27 (0.26–0.29)			
	**Control**	**(*n* = 96)**	**(*n* = 4)**			
	Mean ± SD.	1.10 ± 0.17	1.17 ± 0.20	U = 127.50	0.268	r = 0.115 (−0.084–0.304)
	Median (IQR)	1.10 (0.93–1.20)	1.26 (1.07–1.28)			

SD: Standard deviation; U: Mann-Whitney test; IQR: Interquartile range; t: Student *t*-test; *p*: *p*-value for comparing between different categories; CI: confidence interval; *: Statistically significant at *p* < 0.05. After applying the Benjamini–Hochberg false discovery rate (FDR) correction, all statistically significant associations remained significant.

## Data Availability

Upon reasonable request, the corresponding author will provide the datasets that support the results of this work.

## Supplementary Materials

The following supporting information can be downloaded at: https://www.mdpi.com/article/10.3390/ijms27219617/s1, Table S1: Diagnostic performance for TFAM mRNA level and mtDNA CN for discriminating MI patients (n = 100) from controls (n = 100) after bootstrap; Table S2: Comparison of Firth regression with conventional maximum likelihood core models; Table S3: Complete expanded cardiometabolic genotype model; Table S4: Allele Frequency and Genotype Distribution for *TFAM* rs1937 (G>C) across different populations from the ALFA dataset (NCBI dbSNP rs1937); Table S5: Minor allele frequency of *TFAM* rs1937 variant in different populations from the 1000 Genomes dataset (NCBI dbSNP rs1937); Figure S1: It shows number of participants who were screened, excluded, genotyped and included in the final analysis of the study.
