# Supplementary material for: TFAM Expression, rs1937 Variant, and Mitochondrial DNA Copy Number in Egyptian Myocardial Infarction Patients
Source: Int J Mol Sci. 2026 Oct 27;27(21):9617. doi: 10.3390/ijms27219617 (PMC13763311; doi:10.3390/ijms27219617)
Supplement: Supplementary file 1 [file ijms-27-09617-s001.zip › ijms-4576084-supplementary.pdf]

Supplementary table S1: Diagnostic performance for TFAM mRNA level and mtDNA CN for discriminating MI patients (n = 100) from controls (n = 100) after bootstrap.

Marker	Apparent AUC	Bootstrap 95% CI	CV sensitivity	CV specificity
***TFAM* mRNA expression**	0.99815	0.99490–1.00000	97.0%	100.0%
**mtDNA CN**	0.99500	0.98835–0.99905	93.0%	100.0%

AUC: area under the curve; CI: confidence interval; mtDNA CN: mitochondrial DNA copy number

Supplementary table S2: Comparison of Firth regression with conventional maximum likelihood core models.

Exposure	MLE OR (Wald 95% CI)	MLE p	Firth OR (profile 95% CI)	Firth p
GC genotype (rs1937)	7.095 (1.958 to 25.709)	0.003*	6.263 (1.965 to 23.887)	0.001*
TFAM per 0.1 unit	3.33e-04 (4.15e-07 to 0.267)	0.019*	0.013 (7.25e-05 to 0.098)	<0.001*
mtDNA CN per 0.1 unit	0.002 (3.40e-06 to 0.819)	0.043*	0.036 (3.10e-04 to 0.152)	<0.001*

OR: odds ratio; CI: confidence interval; MLE: maximum likelihood estimation; mtDNA CN: mitochondrial DNA copy number; p: p-value for comparing between different categories; *: Statistically significant at $P < 0.05$.

Supplementary table S3: Complete expanded cardiometabolic genotype model.

Predictor	OR (95% profile CI)	p
**GC genotype (rs1937)**	2.536 (0.284 to 16.743)	0.370
**Age per 10 years**	1.195 (0.609 to 2.417)	0.605
**Male**	1.049 (0.275 to 4.261)	0.945
**BMI per kg/m2**	1.105 (0.835 to 1.518)	0.494
**Smoking**	1.570 (0.483 to 5.014)	0.446
**Hypertension**	70.855 (7.684 to 9682.434)	<0.001*
**Diabetes**	40.357 (3.134 to 5821.847)	0.002*
**LDL-c per 10 mg/dL**	2.179 (1.694 to 2.956)	<0.001*
**HDL-c per 10 mg/dL**	0.713 (0.245 to 1.967)	0.518
**Triglycerides per 10 mg/dL**	0.768 (0.550 to 1.065)	0.112

Age is reported per 10 years and BMI per 1 kg/m^2. Sex is male versus female; smoking is yes versus no. Hypertension is yes versus no. Diabetes mellitus is yes versus no. Lipid increments are 10 mg/dL. LDL-c: low density lipoprotein cholesterol; HDL-c: high density lipoprotein cholesterol; BMI: body mass index; p: p-value for comparing between different categories; *: Statistically significant at $P < 0.05$.

Supplementary table S4: Allele Frequency and Genotype Distribution for *TFAM* rs1937 (G>C) across different populations from the ALFA dataset (NCBI dbSNP rs1937)

Population	Sample Size (n)	Ref Allele G	Alt Allele C (MAF)	Ref HMOZ	Alt HMOZ	HTRZ
**Total / Global**	55,890	0.90707	**0.09293**	0.82419	0.01055	0.16575
**European**	40,462	0.89412	**0.10588**	0.80055	0.01231	0.18714
African	6,276	0.97880	0.02120	0.95825	0.00064	0.04111
African Others	220	0.99500	0.00500	0.99091	0	0.00909
African American	4,056	0.97820	0.02180	0.95707	0.00099	0.04227
Asian	186	0.80600	0.19400	0.65591	0.04301	0.30108
East Asian	122	0.82900	0.18000	0.67213	0.03279	0.29508
Other Asian	64	0.78000	0.22000	0.62500	0.06250	0.31250
Latin American 1	588	0.96300	0.03700	0.92857	0.00340	0.06803
Latin American 2	878	0.92000	0.08000	0.84738	0.00683	0.14579
**South Asian**	102	0.92200	**0.07800**	0.84314	0	0.15686

Source: NCBI dbSNP, rs1937 page. https://www.ncbi.nlm.nih.gov/snp/rs1937 (accessed on 13 October 2026)

Supplementary table S5: Minor allele frequency of *TFAM* rs1937 variant in different populations from the 1000 Genomes dataset (NCBI dbSNP rs1937)

Study	Population	Group	Sample Size	Ref Allele	Alt Allele
1000Genomes	Global	Study-wide	5008	G=0.9115	C=0.0885
1000Genomes	African	Sub	1322	G=0.9932	C=0.0068
1000Genomes	East Asian	Sub	1008	G=0.8284	C=0.1716
1000Genomes	Europe	Sub	1006	G=0.9125	C=0.0875
1000Genomes	South Asian	Sub	978	G=0.888	C=0.112
1000Genomes	American	Sub	694	G=0.909	C=0.091
1000Genomes_30X	Global	Study-wide	6404	G=0.9169	C=0.0831
1000Genomes_30X	African	Sub	1786	G=0.9944	C=0.0056
1000Genomes_30X	Europe	Sub	1266	G=0.9171	C=0.0829
1000Genomes_30X	South Asian	Sub	1202	G=0.8943	C=0.1057
1000Genomes_30X	East Asian	Sub	1170	G=0.8282	C=0.1718

Source: NCBI dbSNP, rs1937 page. https://www.ncbi.nlm.nih.gov/snp/rs1937 (accessed on 13 October 2026)

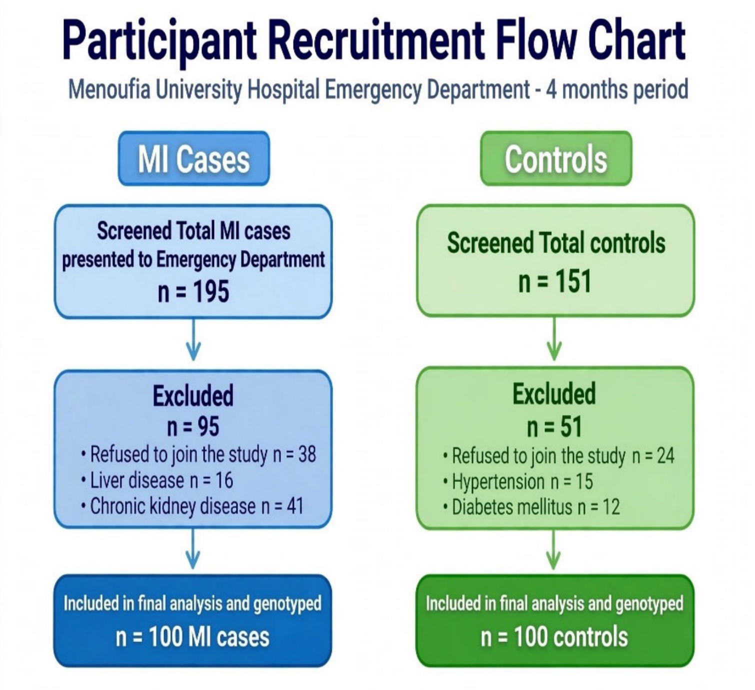

Supplementary Figure S1: It shows number of participants who were screened, excluded, genotyped and included in the final analysis of the study.
